# Large defect closure using a helix tacking system and endoclips after endoscopic submucosal dissection of two adjacent colonic lesions

**DOI:** 10.1055/a-2318-3282

**Published:** 2024-05-29

**Authors:** Elena De Cristofaro, Pierre Lafeuille, Jérôme Rivory, Jeremie Jacques, Rosario D'Almeida, Alexandru Lupu, Mathieu Pioche

**Affiliations:** 1 60259 Gastroenterology, University of Rome Tor Vergata Faculty of Medicine and Surgery, Rome, Italy; 2 36609 Gastroenterology and Endoscopy Unit, Hôpital Edouard Herriot, Lyon, France; 3 37925 Gastroenterology and Endoscopy Unit, CHU Dupuytren, Limoges, France

Endoscopic resection of gastrointestinal neoplasia, especially endoscopic submucosal dissection (ESD), is increasingly common, but poses risks of complications including bleeding and perforation, which can be prevented by closing the defect. Conventional through-the-scope (TTS) clips are typically effective for successful closure of linear defects up to 2 cm in diameter 1 . For larger defects over-the scope (OTS) clips can be used, but incorrect placement complicates the use of other devices. The OverStitch OTS suturing system can achieve full-thickness closure 2 . Nevertheless, it can be used only with a gastroscope, which must be removed to assemble the device. The X-Tack Endoscopic HeliX Tacking System is a novel TTS device designed for endoscopic approximation of soft tissue through a standard gastroscope or colonoscope 3 . Several case reports have demonstrated its efficacy in closing large mucosal defects 4 5 . Herein, we present the combined use of the X-Tack with TTS clips to close a large defect following ESD ( Video 1 ).

Combined use of the X-Tack and through-the-scope clips to close a large defect following endoscopic submucosal dissection.Video 1

A 69-year-old man with ulcerative colitis was referred to our center for the resection of two adjacent flat lesions in the sigmoid colon. ESD of each lesion was performed, with use of an adaptive traction system (ATRACT-2) to better expose the submucosa during the dissection. Given the large defect that resulted from the resection of these two adjacent lesions, closure was performed using the X-Tack and TTS clips in combination: four tacks were placed in a “Z” pattern and two TTS clips were placed at the edges to ensure complete closure ( Fig. 1 ). No adverse events were reported.

**Fig. 1:**
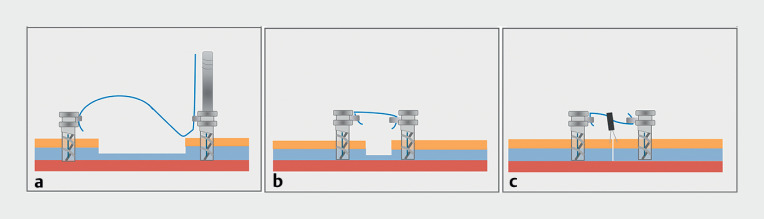
Schematic representation of the combined X-Tack and endoclips strategy illustrating: **a** placement of tacks in healthy tissue adjacent to the tissue defect; **b** tightening of the coaxial suture; **c** placement of through-the-scope clips to fully close the defect.

We can infer that the combined use of TTS clips and the X-Tack can be beneficial for achieving complete closure of large defects after endoscopic resection. This system is relatively straightforward to use, eliminating the need for endoscope withdrawal for device loading, and can be employed when single TTS clips prove inadequate.

Endoscopy_UCTN_Code_TTT_1AO_2AO

## Notes

Endoscopy E-Videos https://eref.thieme.de/e-videos
*E-Videos* is an open access online section of the journal *Endoscopy* , reporting on interesting cases and new techniques in gastroenterological endoscopy. All papers include a high-quality video and are published with a Creative Commons CC-BY license. Endoscopy E-Videos qualify for HINARI discounts and waivers and eligibility is automatically checked during the submission process. We grant 100% waivers to articles whose corresponding authors are based in Group A countries and 50% waivers to those who are based in Group B countries as classified by Research4Life (see: https://www.research4life.org/access/eligibility/ ). This section has its own submission website at https://mc.manuscriptcentral.com/e-videos .
